# Extracellular vesicles in heart failure – A study in patients with heart failure with preserved ejection fraction or heart failure with reduced ejection fraction characteristics undergoing elective coronary artery bypass grafting

**DOI:** 10.3389/fcvm.2022.952974

**Published:** 2022-10-18

**Authors:** Dmitri Matan, Fariborz Mobarrez, Ulrika Löfström, Matthias Corbascio, Mattias Ekström, Camilla Hage, Patrik Lyngå, Bengt Persson, Maria Eriksson, Cecilia Linde, Hans Persson, Håkan Wallén

**Affiliations:** ^1^Division of Cardiovascular Medicine, Department of Clinical Sciences, Karolinska Institutet, Danderyd University Hospital, Stockholm, Sweden; ^2^Heart and Vascular Theme, Karolinska University Hospital, Stockholm, Sweden; ^3^Division of Clinical Chemistry, Department of Medical Sciences, Uppsala University, Uppsala, Sweden; ^4^Department of Medicine, Capio St. Göran Hospital, Stockholm, Sweden; ^5^Department of Medicine, Karolinska Institutet, Stockholm, Sweden; ^6^Department of Molecular Medicine and Surgery, Karolinska Institutet, Stockholm, Sweden; ^7^Department of Cardiology, Danderyd Hospital, Stockholm, Sweden; ^8^Department of Clinical Science and Education, Södersjukhuset, Stockholm, Sweden; ^9^Science for Life Laboratory, Department of Cell and Molecular Biology, Uppsala University, Uppsala, Sweden

**Keywords:** extracellular vesicles, heart failure, Connexin-43, Caveolin-3, troponin-T, myeloperoxidase, Pentraxin-3, VE-cadherin

## Abstract

**Aims:**

Extracellular vesicles (EVs) were investigated as potential biomarkers associated with heart failure (HF) pathophysiology in patients undergoing elective coronary artery bypass surgery characterized by HF phenotype.

**Materials and methods:**

Patients with preoperative proxy-diagnoses of HF types i.e., preserved (HFpEF; *n* = 19) or reduced ejection fraction (HFrEF; *n* = 20) were studied and compared to patients with normal left ventricular function (*n* = 42). EVs in plasma samples collected from the coronary sinus, an arterial line, and from the right atrium were analyzed by flow cytometry. We studied EVs of presumed cardiomyocyte origin [EVs exposing Connexin-43 + Caveolin-3 (Con43 + Cav3) and Connexin-43 + Troponin T (Con43 + TnT)], of endothelial origin [EVs exposing VE-Cadherin (VE-Cad)] and EVs exposing inflammatory markers [myeloperoxidase (MPO) or pentraxin3 (PTX3)].

**Results:**

Median concentrations of EVs exposing Con43 + TnT and Con43 + Cav3 were approximately five to six times higher in coronary sinus compared to radial artery indicative of cardiac release. Patients with HFrEF had high *trans*-coronary gradients of both Con43 + TnT and Con43 + Cav3 EVs, whereas HFpEF had elevated gradients of Con43 + Cav3 EVs but lower gradients of Con43 + TnT. Coronary sinus concentrations of both Con43 + TnT and Con43 + Cav3 correlated significantly with echocardiographic and laboratory measures of HF. MPO-EV concentrations were around two times higher in the right atrium compared to the coronary sinus, and slightly higher in HFpEF than in HFrEF. EV concentrations of endothelial origin (VE-Cad) were similar in all three patient groups.

**Conclusion:**

Con43 + TnT and Con43 + Cav3 EVs are released over the heart indicating cardiomyocyte origin. In HFrEF the EV release profile is indicative of myocardial injury and myocardial stress with elevated *trans*-coronary gradients of both Con43 + TnT and Con43 + Cav3 EVs, whereas in HFpEF the profile indicates myocardial stress with less myocardial injury.

## Introduction

Heart failure (HF) is a severe and common condition with high cost to society (1, 2). Since 2016 ESC guidelines define three phenotypes by left ventricular (LV) ejection fraction (EF): HF with reduced EF (HFrEF; ≤40%), HF with mildly reduced EF (HFmrEF; 41–49%), and HF with preserved EF (HFpEF; ≥50%) with later refinements in the Universal HF definition (3). Given the clinical and mechanistic complexity of HF, biomarkers which may carry pathophysiological information would be of great value to increase the understanding of mechanisms and how they may differ by HF phenotype (4). Extracellular vesicles (EVs) and their potential use as biomarkers in cardiovascular diseases have been studied for several years (5). They are released from virtually all cell types and are, based on their mechanism of generation and size, divided into three groups: exosomes, microvesicles (or microparticles), and apoptotic bodies. EVs carry and expose various molecules and are important for intercellular communication (5). Depending on their cargo, they may give information on various biological events and pathophysiologies (6). Still, information is limited regarding EVs generated and released from the human heart (5), especially in HF. We therefore investigated EVs released from the heart through assessment of *trans*-coronary EV concentration gradients in a group of patients commonly having disturbances in LV function, i.e., patients with stable coronary artery disease (CAD) undergoing elective bypass surgery (CABG). The study patients were divided into three HF phenotypes according to EF, see below.

## Materials and methods

### Study design and heart failure phenotyping

This is an exploratory sub-study of the CABG PREFERS study (7) which included patients with stable CAD without concomitant valvular disease or cardiomyopathy planned to undergo elective CABG surgery at the Karolinska University Hospital between January 2014 and June 2017. The overall aim of the PREFERS studies are to identify new biomarkers to find pathophysiological mechanisms for HFpEF (7). In the present study a subset of patients in CABG PREFERS were included to study EVs in plasma focusing on *trans*-coronary concentration gradients. The patients underwent clinical evaluation, blood sampling for routine blood tests, electrocardiogram (ECG), and transthoracic echocardiography prior to surgery. According to the results of echocardiography and NT-proBNP concentrations, patients were divided into three phenotypes with respect to EF: i.e., HFpEF, (EF ≥ 45% and objective criteria for HFpEF), HFrEF (EF < 45%), or normal LV function, according to a previously described algorithm (8). As not all patients had clinical symptoms of HF at inclusion, the HF diagnoses presented herein should be regarded as proxy-diagnoses of HF, but are named HFpEF, HFrEF, or normal LV function as measured by Doppler echocardiography (in the following called “Normal”). The definition of HF phenotypes reflects the time the study was planned which was before the addition of the HFmrEF phenotype.

Patients were further divided into three groups based on the extent of CAD on the preoperative coronary angiography: left main stenosis (LMS), three-vessel disease (3-VD), and one- or two-vessel disease (1- or 2-VD) (9, 10).

See Supplementary data for more details on echocardiography and HF phenotyping.

### Coronary artery bypass surgery procedure and blood sampling for extracellular vesicles determination

Anesthesia was induced with propofol and fentanyl, further maintained with sevoflurane in oxygen/air, additional doses of fentanyl, and subsequently a continuous propofol infusion during ECC. Muscle relaxation was achieved with atracurium or rocuronium before endotracheal intubation.

All patients were in a fasting state. Blood sampling for EV measurement was performed during the initial phase of the surgical procedure before cardiopulmonary bypass and before entering extracorporeal circulation and heparinization. Blood was sampled using a syringe and carefully drawn from an indwelling catheter in the radial artery, from the right atrium through a central venous catheter, and from the coronary sinus through a 12 French retrograde catheter. Blood samples were then immediately dispensed in a tube containing sodium citrate and centrifuged at 20°C at 2,000 × *g* for 15 min to obtain platelet poor plasma. After centrifugation, samples were loaded on a Hamilton Microlab STARlet liquid handling robot (Hamilton Robotics AB, Kista, Sweden) and aliquoted into 300 μL REMP tubes (Brooks Life Sciences, Chelmsford, MA, USA) uniquely labeled with QR codes. Aliquoted REMP tubes were foil-sealed with a Brooks CSP8 sealer (Brooks Life Sciences, as above) and immediately stored at −80°C. Time from blood sampling to −80°C freezer was <2 h.

### Definitions and analysis of extracellular vesicles

We defined EVs measured by flow cytometry as vesicles between ∼0.3 and 0.9 μm (previously known as microvesicles/particles). Differences in EV concentrations between plasma samples from *sinus coronarius* and *radial artery* were calculated and are expressed as *trans*-coronary gradients (i.e., delta-values; unity vesicles/μL). A positive *trans*-coronary concentration gradient can indicate release of EVs over the heart and/or a reduced EV clearance, a negative gradient can indicate reduced release and increased uptake/and or degradation of EVs over the heart, and/or release of EVs from the lung tissue/pulmonary vasculature.

Fluorescent labeled antibodies toward Connexin-43 (11), Troponin T (TnT), and Caveolin-3 (12) were used to detect EVs that could be of cardiomyocyte origin; we measured EVs co-exposing Connexin-43 and TnT (Con43 + TnT EVs) or Connexin-43 and Caveolin-3 (Con43 + Cav3 EVs).

As an additional potential cardiomyocyte marker we measured EVs exposing N-Cadherin (N-Cad) (13).

To study the vascular endothelium EVs exposing VE-cadherin (VE-Cad) were measured (14).

For inflammation EVs exposing myeloperoxidase (MPO) (15–17) or Pentraxin-3 (PTX3) (18) were studied.

See Supplementary Figures 2, 3, and Supplementary material, for further details.

### Statistical analysis

Continuous variables are expressed as mean value and standard deviation (SD), or median value and interquartile range (IQR) or percentiles, as appropriate, while categorical variables are expressed with number of cases (*n*) and/or proportions. The following statistical tests have been used: Mann–Whitney test for comparison of two groups of independent variables, Kruskal–Wallis test for comparison of >2 groups of independent variables, and Wilcoxon signed rank test for comparison of two groups of dependent variables. Correlations were studied with Spearman’s rank correlation coefficient. A *p*-value <0.05 was considered statistically significant.

For statistical calculations SPSS software, Version 25 has been used (IBM Corporation, Armonk, NY, USA).

### Ethical considerations

The CABG PREFERS study was approved by the Stockholm Ethical Review Board, Sweden (2013/1869-31/1) and was conducted according to International Conference on Harmonization and Good Clinical Practice guidelines and compliant with the Declaration of Helsinki.

Oral and written informed consent was obtained from all study participants, all included between January 2014 and June 2017.

## Results

The present sub-study included patients where central blood samples were available. For logistic reasons, central blood sampling could not always be performed in all patients included in the CABG PREFERS study. Samples from the coronary sinus and radial artery were available in 81 patients, and for all three sampling locations (coronary sinus, radial artery, and right atrium) in 80 patients.

The median age was 71 years, and 88% were men (Table 1). A large majority of patients (78%) had hypertension, 17% had atrial fibrillation (AF), and 31% had diabetes mellitus. Most patients (84%) had severe angiographic CAD, with three-vessel disease or stenosis of the left main coronary artery (Supplementary Figure 1).

**TABLE 1:** Clinical characteristics of patients with extracellular vesicles measured.

		All patients	Patient category		
			
			HFpEF	HFrEF	Normal
N		81 (100%)	19 (23%)	20 (25%)	42 (52%)
Women		10 (12%)	1 (5%)	1 (5%)	8 (19%)
Age, years		71 (64–75)	72 (68–76)	72 (60–77)	68 (63–75)
BMI, kg/m^2^		27 (24–29)	26 (24–29)	28 (24–33)	27 (25–29)
NT-pro-BNP, ng/L		235 (120–694)	225 (176–604)	976 (615–2,400)	150 (80–269)
LVEF, %		57 (46–61)	58 (56–63)	43 (37–44)	59 (57–62)
Coronary angiography data	1- or 2-VD, no LM stenosis	13 (16%)	7 (37%)	3 (15%)	3 (7%)
	3-VD, no LM stenosis	48 (59%)	10 (53%)	11 (55%)	27 (64%)
	LM stenosis	19 (23%)	2 (11%)	6 (30%)	11 (26%)
	not reported	1 (1%)	0	0	1 (2%)
HT		63 (78%)	17 (90%)	18 (90%)	28 (67%)
AF		14 (17%)	1 (5%)	9 (45%)	4 (10%)
COPD		7 (9%)	1 (5%)	3 (15%)	3 (7%)
Renal failure		13 (16%)	6 (32%)	5 (25%)	2 (5%)
Anemia		22 (27%)	5 (26%)	9 (45%)	8 (19%)
Diabetes Mellitus		25 (31%)	3 (16%)	9 (45%)	13 (31%)
PVD		5 (6%)	3 (16%)	2 (10%)	0
CVD/TIA		2 (2%)	1 (5%)	0	1 (2%)
Previous AMI		10 (12%)	2 (11%)	1 (5%)	7 (17%)

Numbers, proportions (%), and median and IQR (interquartile range) are shown. AF, atrial fibrillation; AMI, acute myocardial infarction; BMI, body mass index; COPD, chronic obstructive pulmonary disease; CVD, cerebrovascular disease; HFpEF, heart failure with preserved ejection fraction; HFrEF, heart failure with reduced ejection fraction; HT, hypertension; LM, left main coronary artery; LVEF, left ventricular ejection fraction; NT-pro-BNP, N-terminal pro-B-type natriuretic peptide; PVD, peripheral vascular disease; TIA, transient ischemic attack; VD, (coronary artery) vessel disease. Data on HT, AF, COPD, Diabetes, PVD, CVD/TIA, and previous AMI were based on ICD codes.

Around 70% of patients were on antiplatelet agents and 12% on oral anticoagulants. More than 60% of patients were on beta-adrenoreceptor antagonists, and 81% on statin therapy. Less than 1/3 of patients had other HF medication, such as angiotensin-converting enzyme inhibitors, angiotensin II-receptor antagonists and/or mineralocorticoid receptor antagonists. Seven percent of patients were on diuretics (furosemide), and this was equally distributed between HF phenotypes.

See Supplementary Table 2 for details on pharmacological treatment.

### Extracellular vesicles in all patients

**FIGURE 1:**
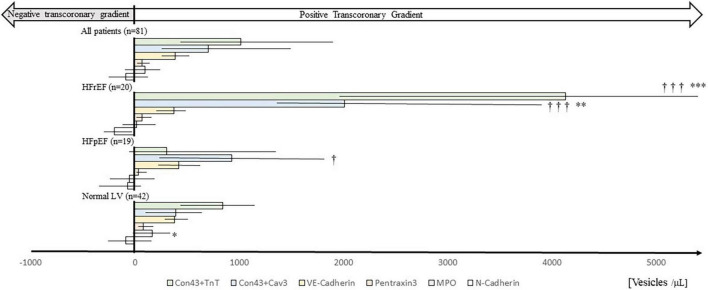
*Trans*-coronary gradients of extracellular vesicles in patients presented by heart failure phenotype. Horizontal bars show median *trans*-coronary concentration gradients of different EV phenotypes in patients with HFrEF, HFpEF, or Normal LV phenotype calculated as plasma concentrations in coronary sinus *minus* concentrations in radial artery. For comparison all patients (*n* = 81), irrespective of HF phenotype, are shown (top of figure). Median values (bars), interquartile ranges (horizontal lines), and *p*-values (between patient group comparisons; Mann–Whitney *U*-test) are also shown. *p < 0.05, ^**^p < 0.01, ^***^p < 0.001 compared to HFpEF. ^†^*p* < 0.05, ^†††^*p* < 0.001 compared to Normal. Con43 + TnT: Connexin-43 + Troponin T; Con43 + Cav3: Connexin-43 + Caveolin 3; MPO: Myeloperoxidase. Numerical data are shown in Supplementary Table 3.

**FIGURE 2:**
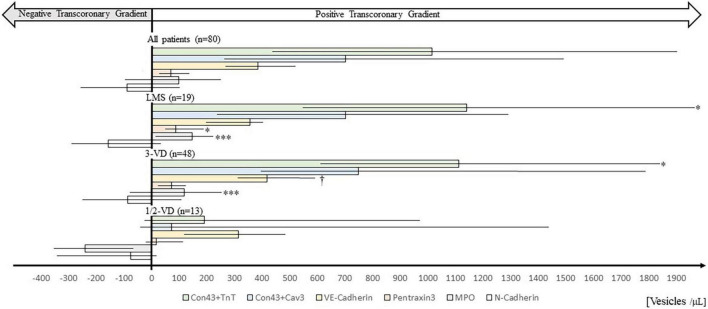
*Trans*-coronary gradients of extracellular vesicles in patients presented by extent of coronary artery disease based on preoperative coronary angiography. Horizontal bars show median trans-coronary EV concentration gradients, calculated as plasma concentrations in coronary sinus minus plasma concentrations in radial artery. For comparison all patients (n 80) irrespective of disease severity are shown (top of figure). CAD angiography data was missing in one patient. Median values (bars), interquartile ranges (horizontal lines), and p-values (between patient group comparisons; Mann-Whitney U-test) are shown. *p < 0.05, ^***^p < 0.001 compared to 1/2-VD. ^†^p 0.05 compared to LMS. 1/2-VD, one or two-vessel disease; 3-VD, three-vessel disease; LMS, stenosis of the left main coronary artery. Con43 + TnT, Connexin-43 + Troponin T; Con43 + Cav3, Connexin-43 + Caveolin 3; MPO, Myeloperoxidase. Numerical data are shown in Supplementary Table 4.

We found positive *trans*-coronary gradients for Con43 + TnT and Con43 + Cav3 EVs indicating cardiac release. There were positive but much smaller *trans*-coronary gradients also for VE-Cad EVs, PTX3 EVs, and MPO EVs, while for N-Cad EVs the *trans*-coronary concentration gradients were negative suggesting uptake or degradation of EVs over the heart, or perhaps a small release of N-Cad EVs from the lung tissue/pulmonary vasculature. Transcoronary gradients for all extracellular vesicles are shown in top panel of Figures 1, 2.

### Extracellular vesicles in patients divided by heart failure phenotype

Heart failure with reduced ejection fraction patients had the highest *trans*-coronary gradients of Con43 + TnT and Con43 + Cav3 EVs (see Figure 1). The Normal LV group had a Con43 + TnT and Con43 + Cav3 EV profile that resembled HFrEF patients but with lower *trans*-coronary gradients, and the lowest median *trans*-coronary concentration gradient of Con43 + Cav3 EVs. Notably, HFpEF patients had a different *trans*-coronary EV profile compared to HFrEF and Normal physiology, with Con43 + Cav3 EVs as the EV phenotype with the most prominent *trans*-coronary concentration gradient. VE-Cad EV concentration gradients were similar in all three phenotypes. Detailed data on concentrations of the different EV types in samples from coronary sinus and radial artery divided by HF phenotypes are shown in Supplementary Figures 4–8 and also in Supplementary Table 3.

### Extracellular vesicles and echocardiographic parameters and age, sex, and diabetes

As illustrated in Table 2, the concentrations of Con43 + Cav3 EVs in coronary sinus were correlated with preoperative echocardiographic parameters associated with HF: GLS (*r* = 0.51; *p* < 0.001), LVEF (*r* = −0.43; *p* < 0.001), LAVI (*r* = 0.43; *p* < 0.001), E/é (*r* = 0.23; *p* = 0.039), and TR V-max (*r* = 0.30; *p* = 0.044).

**TABLE 2:** Correlations between extracellular vesicles in plasma samples from coronary sinus and preoperative clinical/echocardiographic parameters in all patients, irrespective of heart failure phenotype.

Type of EVs (EV/μ L)		Age	NT-pro-BNP	GLS	LVEF	LVMI	LAVI	E/é	TRV-max
	
	n	81	80	81	81	81	81	81	81
Connexin-43 + Caveolin-3	*p*	0.283	<0.001	<0.001	<0.001	0.154	<0.001	0.039	0.044
	*r*	0.121	0.424	0.511	–0.431	0.169	0.433	0.230	0.301
Connexin-43 + TnT	*p*	0.878	0.022	<0.001	<0.001	0.013	0.021	0.128	0.072
	*r*	–0.017	0.257	0.409	–0.509	0.275	0.255	0.171	0.270
MPO	*p*	0.195	0.093	0.249	0.632	0.182	0.279	0.735	0.162
	*r*	–0.145	−0,189	–0.129	0.054	–0.150	–0.122	–0.038	–0.212
VE-Cadherin	*p*	0.245	0.144	0.826	0.495	0.471	0.169	0.980	0.058
	*r*	0.131	–0.165	–0.025	0.077	0.081	–0.154	0.003	–0.285
N-Cadherin	*p*	0.799	0.056	0.698	0.651	0.978	0.049	0.055	0.780
	*r*	–0.029	–0.214	–0.044	0.051	–0.003	–0.220	0.214	–0.043
PTX3	*p*	0.482	0.549	0.598	0.946	0.481	0.855	0.993	0.692
	*r*	–0.079	–0.068	–0.060	0.008	–0.079	0.021	–0.001	–0.061

Echocardiography was performed 10 ± 7 weeks before surgery. r = Spearman’s rank correlation coefficient. E/é, ratio of mitral Doppler E velocity to mitral tissue Doppler é velocity; GLS, global longitudinal strain; LAVI, left atrial volume index; LVEF, left ventricular ejection fraction; LVMI, left ventricular mass index; MPO, myeloperoxidase; PTX3, Pentraxin-3; TnT, Troponin-T; TRV-max, maximum flow velocity of tricuspid regurgitation.

Also Con43 + TnT EVs and echocardiographic parameters were correlated: GLS (*r* = 0.41; *p* < 0.001), LVEF (*r* = −0.51; *p* < 0.001), LAVI (*r* = 0.25; *p* = 0.021), and LVMI (*r* = 0.28; *p* = 0.013), and there was a correlation of borderline significance between Con43 + TnT EVs and TR V-max (*r* = 0.27; *p* = 0.072).

Furthermore, Con43 + Cav3 as well as Con43 + TnT EVs concentrations correlated significantly with preoperative NT-pro-BNP concentrations (*r* = 0.42; *p* < 0.001 and *r* = 0.26; *p* = 0.022, respectively).

As an additional approach to assess relationships between Con43 + TnT and Con43 + Cav3 EVs and HF related parameters, we divided echocardiographic variables and NT-proBNP in tertiles. As shown in Supplementary Table 1, Con43 + Cav3 EV concentrations increased with increasing NT-proBNP levels and with echocardiographic signs of reduced left atrial and ventricular performance and increased filling pressure, and Con43 + TnT EV concentrations increased with increasing NT-pro-BNP levels and decreasing ventricular performance. Both EV types were associated with increasing indirect measures of LV filling pressure.

Regarding the other EVs no consistent relationships were observed between EV concentrations in coronary sinus, and NT-pro-BNP or echocardiographic parameters.

There were no correlations between EV concentrations in coronary sinus and age. Data on all correlations are shown in Table 2.

EV concentrations were similar in patients with compared to those without diabetes mellitus, and in men compared to women. Concentrations of Con43 + Cav3 and Con43 + TnT EVs were, however, significantly higher in patients with AF compared to those with no AF (Supplementary Table 5).

### Extracellular vesicles in patients divided by coronary artery disease angiography data

Patients with 3VD and patients with LMS had a similar EV profile; median Con43 + TnT EVs concentration gradients were the highest followed by Con43 + Cav3 EVs and VE-Cad EVs. Con43 + TnT EV concentration gradients were significantly higher in patients with 3VD or LMS compared to 1- or 2-VD (*p* < 0.05 for both). In contrast, Con43 + Cav3 EV concentrations did not differ significantly between CAD disease groups, as there was a pronounced inter-patient variability in the concentration gradients of this EV type in patients with 1- or 2-VD (Figure 2 and Supplementary Table 4).

Patients with 3-VD or LMS had significantly higher *trans*-coronary gradients of MPO EVs than those with 1- or 2-VD (*p* < 0.001 for both). In fact, the median concentration gradient for MPO EVs was negative in patients with 1- or 2-VD (Figure 2).

The N-Cad EV concentration gradient was negative and of similar magnitude in all three CAD groups, indicating that these EVs accumulated in the heart and/or that these EV were to some extent released from lung tissue/pulmonary vasculature.

See Supplementary Table 4 for numerical details on EV *trans*-coronary concentration gradients.

### Extracellular vesicle concentrations in coronary sinus, radial artery, and right atrium, in all patients

Median concentrations for Con43 + TnT and Con43 + Cav3 EVs were approximately five to six times higher in coronary sinus compared to the radial artery. The concentration of Con43 + TnT and Con43 + Cav3 EVs in the right atrium was in the same low concentration range as in the radial artery indicating negligible systemic contribution of these EVs.

The median MPO-EV concentration was almost two times higher in the right atrium compared to in the radial artery or coronary sinus, and right atrium concentrations were slightly higher in HFpEF compared to HFrEF [median concentrations 1157 vs. 929 vesicles/μL (*p* = 0.05) in HFpEF and HFrEF, respectively].

PTX3 EV concentrations were also highest in the right atrium, but in lower concentrations and with less pronounced differences between sampling sites compared to what was observed for the MPO EVs (Data on EV concentrations in coronary sinus, radial artery, and right atrium are shown in Table 3).

**TABLE 3:** Concentration in plasma of extracellular vesicles collected from the radial artery, coronary sinus, and right atrium in all patients, irrespective of heart failure phenotype or coronary artery disease severity grading.

Type of EVs (EVs/μ L)	Radial artery (*n* = 80)	Coronary sinus (*n* = 80)	Right atrium (*n* = 80)	*P* *	*P* ^†^	*P* ^††^
Connexin-43 + Caveolin-3	131 (87–168)	849 (354–1,636)	159 (90–222)	< 0.001	< 0.001	0.083
Connexin-43 + TnT	251 (122–360)	1,258 (658–2,194)	132 (72–217)	< 0.001	< 0.001	< 0.001
VE-Cadherin	487 (460–522)	882 (771–1027)	682 (649–736)	< 0.001	< 0.001	< 0.001
Pentraxin-3	346 (298–380)	396 (364–498)	526 (480–561)	< 0.001	< 0.001	< 0.001
MPO	563 (440–760)	650 (574–739)	1,106 (778–1,230)	0.009	< 0.001	< 0.001
N-Cadherin	541 (378–724)	476 (395–540)	672 (636–750)	0.001	< 0.001	< 0.001

Refers to *p*-values for comparison between concentrations in: *coronary sinus and radial artery, ^†^ coronary sinus and right atrium, ^††^ radial artery and right atrium. (Median and IQR; Wilcoxon signed rank test). In one patient samples from the right atrium were missing, thus n = 80. TnT, Troponin T; MPO, myeloperoxidase.

## Discussion

In patients undergoing elective CABG we found that (a) Con43 + TnT and Con43 + Cav3 EV concentrations were elevated in coronary sinus compared to radial artery, (b) the Con43 + TnT and Con43 + Cav3 EV profile was different in patients with HFrEF compared to those with HFpEF, (c) the coronary sinus concentrations of Con43 + TnT and Con43 + Cav3 EVs correlated significantly with echocardiographic and laboratory markers of HF. Our data indicate that the EV phenotype exposing Con43 + TnT and Con43 + Cav3 are released from cardiomyocytes. The EV release associates with markers of HF functional severity.

HFpEF patients were characterized by a positive Con43 + Cav3 EV concentration gradient, and the coronary sinus concentrations of these EVs correlated significantly with NT-pro-BNP levels and echocardiographic parameters reflecting elevated filling pressure. Data from a recent experimental study show that myocytes exposed to cyclic stretching release significant amounts of EVs (19). Based on these findings and our present data, it is reasonable to conclude that human cardiomyocytes release EVs in a stress-dependent manner. Con43 + Cav3 EVs as well as Con43 + TnT EV concentrations in coronary sinus increased with impaired systolic left ventricular performance, further supporting the idea that cardiomyocytes release EVs upon decreasing cardiac function. The release of Con43 + TnT and Con43 + Cav3 EVs was greater in HFrEF patients than in HFpEF or in patients with intact LV function. The most abundant EV type in coronary sinus samples from HFrEF were those exposing Con43 + TnT. The concentration gradients of these EVs were higher in patients with more advanced coronary artery disease. Circulating EVs exposing troponin have also been observed in a murine model of myocardial infarction (20) and should reflect cardiomyocyte necrosis. Troponins exposed on EVs may, however, also emanate from reversibly injured cardiomyocytes which due to mechanical stress and overload release EVs exposing troponins originating from a “rapidly releasable intracellular pool” (21, 22). Supporting this idea circulating EVs carrying troponin have recently been found in patients with severe aortic stenosis (23). The high release of Con43 + TnT EVs found in the HFrEF patients is thus likely a sign of cardiomyocyte turn-over and disease activity of coronary artery disease. It may, however, also reflect increased mechanical stress of the myocardium as shown by significant correlations with several echocardiographic variables reflecting decreased cardiac function. The lower *trans*-coronary gradient of Con43 + TnT EVs found in the HFpEF patients is in line with a recent study where it was shown that circulating troponin was a biomarker for future HFrEF but not for HFpEF, thus supporting our results (4). Measurements of EV-bound troponin with flow cytometry enable phenotyping with additional markers, i.e., measurements of co-expression of several markers of pathophysiological interest simultaneously, which is not possible with conventional assays of circulating troponin. The potential added value of measurements of EV-bound troponin versus measuring total troponin in plasma with conventional assays should be investigated more in detail in future studies. The Con43 + TnT and Con43 + Cav3 EV concentrations in the coronary sinus were high in comparison to the concentrations measured at the arterial level or in the right atrium. This indicates a rapid clearance of these EVs when passing through the pulmonary circulation. Mechanisms through which EVs are cleared from the circulation are unknown but may, as regards the pulmonary circulation, include uptake of EVs in resident alveolar macrophages as shown in experimental studies (24). Since it has been shown that EVs are important in paracrine signaling (5) it is of interest to further investigate the role of cardiomyocyte EVs and its influence on the lungs and in the pulmonary circulation. In this respect, it has been put forward that EVs may exert both harmful and protective effects (25). Of note, Khandagale et al. recently showed that circulating EVs from patients with pulmonary arterial hypertension were able to activate pulmonary endothelial cells and induce *in vitro* angiogenesis (26). Very recently, Anselmo et al. reported that cardiomyocyte derived EVs containing ceramides exerted positive inotropic effects on unstressed cardiomyocytes (23) in patients with aortic stenosis, and elevated cardiomyocyte EVs were associated with a favorable outcome (23), the latter in agreement with other studies suggesting that EVs may carry prognostic information in cardiovascular disease (27). Taken together, abundant data warrant further studies on EVs released from cardiomyocytes, as both potential modulators and mediators of cardiovascular disease and HF.

VE-Cad EV concentrations were significantly elevated in coronary sinus versus radial artery, but the concentration gradients were lower than for the Con43 + TnT and Con43 + Cav3 EVs and independent of HF phenotype, systolic or diastolic LV function variables, or NT-pro-BNP levels. Elevations in circulating VE-Cad EVs are strongly associated with endothelial dysfunction in patients with advanced vascular disease (28). VE-cadherin plasma levels correlate with extent of coronary atherosclerosis when assessed as soluble VE-cadherin by an ELISA method (29). These data in combination with previous data on VE-cadherin expressed in complicated inflamed atherosclerotic plaques (30) indicate that VE-Cad EVs generated during the *trans*-coronary passage reflect atherosclerosis of the coronary arteries. There was a slightly higher cardiac release of VE-Cad EVs in patients with three-vessel disease as compared to those with disease of the left main artery, but the methodology we used for classification of CAD extension used is crude, and not sensitive in assessing the quantitative extent of coronary atherosclerosis. This limits the possibility to use endothelial derived EVs to grade coronary atherosclerosis, but this was not the aim of the study.

As regards inflammation we measured EVs exposing PTX3, a molecule locally produced in various cells like cardiomyocytes (31), endothelial cells, macrophages, smooth muscle cells, and fibroblasts (32). Interestingly, an elevated production of PTX3 in the heart has been reported in patients with HFpEF (33). We found positive albeit small *trans*-coronary gradients of PTX3, but with similar magnitudes in HFpEF, HFrEF, and intact LV function. There was a slightly higher gradient in patients with left main disease compared to 1- or 2-VD, but *trans*-coronary gradients and PTX3 EV concentrations in coronary sinus were much lower than those observed for Con43 + TnT or Con43 + Cav3 EVs. There were significantly higher concentrations of PTX3 EVs in right atrium compared to coronary sinus, which indicates that extracardiac sources contributes significantly to the PTX3 EV levels detected in the circulation. This was even more obvious for MPO EVs where median levels were almost two-fold higher in the right atrium compared to coronary sinus. The significant systemic contribution of PTX3 and MPO EVs, as assessed by sampling from the right atrium, may be due to the ongoing surgery. Of further interest is the finding that the MPO-EV concentrations in the right atrium were around 20% higher in HFpEF than in HFrEF, in agreement with the idea that HFpEF is associated with a more pronounced systemic inflammation, but these data should be interpreted with caution since the findings were of border-line significance. There was, however, a statistically significant difference in *trans*-coronary gradients for MPO EVs in relation to CAD severity. We found a higher gradient for MPO EVs in 3-VD or LMS compared to patients with 1- or 2-VD in which the median *trans*-coronary gradient in fact was negative, indicating accumulation of these EVs when passing the heart. Together these findings fit well with studies showing that elevations in circulating MPO reflect the extent of complicated coronary atherosclerotic lesions (15) with ongoing inflammation in the coronary vasculature and myocardial tissue. Patients with more advanced coronary disease may thus have a higher inflammatory burden and our findings raises the question, if the degree of coronary artery disease is secondary to inflammation.

The observed differences in *trans*-coronary EV concentration gradients between the HF phenotypes should be interpreted with some caution. We could not correct for blood flow at the sampling sites. HFpEF is associated with a reduced coronary flow reserve (34, 35) and impaired blood flow over the myocardium may reduce the wash-out of EVs in such patients. This could in turn influence concentrations of EVs down-stream in the organ investigated, as in our case when sampling is performed in the coronary sinus. On the other hand, there are studies reporting a substantially higher coronary sinus blood flow under resting conditions in HFpEF patients as compared to patients without HFpEF (36). If sampling during surgery is more comparable to the resting situation than the hyperemic situation in which a reduced coronary flow reserve has been experimentally revealed (35, 36), an underlying dysfunction of the coronary microcirculation may not impact the *trans*-coronary passage of EVs significantly under our study conditions. The local myocardial uptake and/or degradation of the EVs may also influence *trans*-coronary gradients. In this respect it is of interest to note that we found negative *trans*-coronary gradients of N-Cad EVs in all patient groups studied. This should reflect increased uptake and/or degradation by the heart and the local coronary microcirculation of this EV type, but a release of N-Cadherin EVs when passing the lungs is also possible. The turnover and destiny of EVs is likely complex. It may depend on many factors such as the blood flow, the type of EV, and its surface interactome, the vascular bed and/or organ through which the EVs are passing, as well as disease-associated pathophysiology of the organ.

### Limitations

We studied patients with stable CAD undergoing CABG and our findings can only be related to patients with prevalent ischemic heart disease. The patients were in a stable condition which may not reflect the common clinical HF situation. The study is small and there were few women included. The HF diagnosis and HF phenotyping were based on an algorithm of LV function (37), and thus not a best practice clinical diagnosis. Further, a group of patients with intact LV diastolic function and EF ≥ 45% was identified as having an intact LV function and patients with reduced EF < 45% were classified as HFrEF. The study has an exploratory design and therefore we did not correct *p*-values for multiple comparisons. To study possible EV release from cardiomyocytes we assessed EV exposing molecules that are highly abundant in cardiomyocytes, i.e., Caveolin-3, troponin T and Connexin-43, but these are not entirely specific for cardiomyocytes. Although we found clear-cut positive *trans*-coronary gradients of these EVs phenotypes, our data should be viewed upon as hypothesis generating.

It can be deduced from the data that the Con43 + TnT and Con43 + Cav3EVs have a rapid clearance as the concentrations were significantly lower in blood from the radial artery compared to in blood sampled from the coronary sinus. Studies performed on these EVs in peripheral venous blood from patients with significant HF are needed to further investigate, if they may be suitable as biomarkers in HF. It should be put forward that we studied TnT exposed on the membrane of circulating Con43 + EVs. The TnT molecules may emanate from the cytosol of the cardiomyocyte EV mother cells and bound and exposed to the myocyte cell membrane during budding, but they may also be taken up from plasma and bound to EVs as various proteins present in plasma may adsorb to EVs (38). TnT may therefore be bound to other EVs than those exposing Con43. This deserves to be investigated in future studies.

We measured EV levels in blood samples taken during cardiac surgery, a highly invasive procedure, where different interventions performed during the surgery may have influenced our results. We measured EVs in arterial blood from the radial artery and not from the aortic root. The time for blood constituents to pass from the ascending aorta to the radial artery is, however, very short (seconds) and it is unlikely that this passage should influence EV levels significantly.

## Conclusion

Con43 + TnT and Con43 + Cav3 EVs are released over the heart and associate with HF and signs of myocardial stress, suggesting involvement of stretch-activated pathways leading to cardiomyocyte EV release. The *trans*-coronary EV release profile is different between HF phenotypes with HFrEF being associated with a pronounced release of EVs reflecting myocardial injury. In HFpEF, on the other hand, there is a clear stress-induced release of EVs but much lower release of myocardial injury-related EVs. Con43 + TnT and Con43 + Cav3 EVs are rapidly cleared over the pulmonary circulation, likely due to uptake and degradation in the pulmonary vasculature. As EVs may transfer biological information and have distant effects, future studies on EVs released from cardiomyocytes and its effects on the cardiopulmonary system in HF, as well as other types of cardiovascular diseases, are warranted.

## Data availability statement

The raw data supporting the conclusions of this article will be made available by the authors, without undue reservation.

## Ethics statement

The studies involving human participants were reviewed and approved by Stockholm Ethical Review Board, Sweden. The patients/participants provided their written informed consent to participate in this study.

## Author contributions

HW, FM, and HP: conceptualization. HW, FM, HP, MC, DM, and MEk: formal analysis and methodology. HW, HP, FM, and DM: investigation, validation, visualization, and wrote manuscript—original draft preparation. HW, HP, FM, MEk, DM, CH, CL, UL, MKr, MC, PL, and BP: review and editing of manuscript. All authors contributed to the article and approved the submitted version.
