# Supplementary material for: Extracellular vesicles in heart failure – A study in patients with heart failure with preserved ejection fraction or heart failure with reduced ejection fraction characteristics undergoing elective coronary artery bypass grafting
Source: Front Cardiovasc Med. 2022 Oct 18;9:952974. doi: 10.3389/fcvm.2022.952974 (PMC9622760; doi:10.3389/fcvm.2022.952974)
Supplement: Supplementary file 1 [file Data_Sheet_1.docx]

**Supplementary Data**

**Methods**

*HF phenotyping*
Diagnosis of HFpEF in PREFERS and HFpEF-proxy in CABG PREFERS was based on the presence of LVEF >45% in combination with echocardiographic signs of diastolic and/or structural LV dysfunction and/or elevated NT-pro-BNP levels as previously specified (1). The present and recent ESC guidelines advocate the use of at least 4 up to 8 parameters of structural LV changes and diastolic dysfunction for diagnosis and risk prediction of HFpEF, some of these parameters may be used interchangeably (2). In summary, the number of altered variables may increase the accuracy of the HFpEF diagnosis and risk prediction (3). In the CABG PREFERS study we therefore used state-of-the-art guideline criteria for HFpEF and a majority of the criteria achieved in an individual patient should be positive for rendering a HFpEF-proxy diagnosis.

In the PREFERS study the inclusion criteria were based on symptoms and signs of HF and increase of NT-proBNP in all patients regardless of EF and E/e` >8 in patients with EF ≥45%. These criteria are sensitive and will allow further categorization into HFrEF and more specific groups of HFpEF based on the majority principle as stated above for the number of criteria achieved in an individual patient. To strengthen diagnostic definition in the CABG-PREFERS study we used a previously described consensus method in patients with ambiguous results (3, 4) and allowed for future classification and phenotyping of both PREFERS and CABG PREFERS patients according to the HFpEF definitions in the most recent guidelines by the use of our extensive echocardiographic protocols.

*Echocardiography*

Transthoracic doppler echocardiography was carried out as previously described (1). Two-dimensional evaluation of cardiac chambers regarding size, structure and function, and evaluation of Doppler recordings for diastolic LV function and valvular function were performed according to the American Society of Echocardiography and the European Association of Cardiovascular Imaging recommendations (5, 6). Echocardiographic variables of special interest in the study, except of LVEF, were: global longitudinal strain (GLS, %) as a measure of LV systolic performance, left ventricular mass index (LVMI) indicating LV hypertrophy, left atrial volume index (LAVI) and ratio of mitral Doppler E velocity to mitral annular (tissue Doppler) é velocity (E/é) as variables of diastolic LV function, and maximal velocity of tricuspid regurgitation (TR Vmax) as an indicator of pulmonary arterial pressure.

LVEF was calculated according to the biplane method of disks (modified Simpson's rule). LV mass was calculated from 2D LV linear dimensions using ASE-recommended formula, and the left atrium volume was calculated by biplane method of disks (5). LV GLS was analyzed on 2D images using speckle-tracking and calculated by Automated Functional Imaging (AFI) available on an EchoPAC workstation (GE EchoPAC sw only, Norway). Transthoracic echocardiography was performed using a Vivid 9 ultrasound system (Vingmed-General Electric, Horten, Norway) and all images were digitally stored in a dedicated database. All data analysis and calculations were performed on an EchoPAC workstation by an experienced sonographer. The mean value of three cardiac cycles was calculated for each variable.
A 12-lead ECG was recorded at the baseline inclusion visit before CABG surgery.

*Flow cytometry analysis*
Samples containing platelet poor plasma were thawed in a waterbath at 37 C° for 5 minutes. After thawing, samples were transferred from the REMP tubes into new sample tubes. Samples were then re-centrifuged at 2 000 g for 20 minutes at room temperature (RT) to discard any larger debris and/or artefacts. The upper supernatant was again transferred to new tubes and centrifuged at 20 800 g for 45 minutes at RT. The supernatant, which contains smaller EVs (previously known as exosomes) was discarded (roughly 2/3), and the EV-enriched pellet (previously known as microvesicles/particles) was resuspended in the remaining plasma and used for the flow cytometric analysis. Subsequently, the EV-enriched pellet was divided into new flow cytometric tubes. All antibodies used in the present study were self-conjugated according to manufacturer instructions (Abcam, Cambridge, UK) using the DyLight fluorescent dyes. Briefly, 20 µL of the EV-enriched pellet was incubated for 20 min in dark, with 5 µl anti-Connexin-43 Dylight 488 (final conc. 2.5 µg/ml, Abcam, Cambridge, UK), anti- Caveolin-3 Dylight 633 (final conc. 2.5 µg/ml, Abcam, Cambridge, UK), anti-TnT Dylight 755 (final conc. 2.5 µg/ml, Abcam, Cambridge, UK), anti-VE-Cadherin Dylight 633 (final conc. 2.5 µg/ml, Abcam, Cambridge, UK), anti-MPO Dylight 488 (final conc. 2.5 µg/ml, Abcam, Cambridge, UK), and anti-Pentraxin 3 Dylight 755 (final conc. 2.5 µg/ml, Abcam, Cambridge, UK) and Anti-N-Cadherin Dylight 633 final conc. 2.5 µg/ml, Abcam, Cambridge, UK). The final volume of each tube was set to 150 µl by adding appropriate volume of Sheat fluid (Beckman coulter, Brea, CA, USA).

EVs were measured using a Beckman Gallios flow cytometer (Beckman coulter, Brea, CA, USA) with the threshold set to forward scatter. The EV gate (Supplementary Figure 2) was determined using Megamix-Plus FSC beads (0.3, 0.5 and 0.9 µm in size; BioCytex, Marseille, France). The threshold was set to FSC and the flow rate was set to medium. EVs were defined by size and complexity (less than 0.9 µm in diameter) and positive for antibodies described above, see supplementary Figure 2 for details regarding FCM interpretation. The lower limit of the instrument is around 0.3 µm (forward scatter). Representative flow cytometric plots (including gating) are shown in supplementary Figure 2. Conjugate isotype-matched immunoglobulin with no reactivity against human antigens (anti mouse; all at 2.5 μg/mL) was used as a negative control to define the background noise of the cytometric analysis (Beckman coulter, Brea, CA, USA). In addition, fluorescent minus one (FMO) controls were also used, as well as buffer and unstained samples. Unstained controls were measured at the same dilution as matched stained and isotype controls. Flow cytometer acquisition settings were maintained for all samples, including triggering threshold, voltages, and flow rate. Results are presented as EVs/μL plasma, processed from the 20 μL EV-enriched pellet obtained after high-speed centrifugation (including the dilution factor). The intra- and inter-assay coefficients of the flow cytometric analysis were less than 9.0 % respectively.

**Supplementary Figure 1. Coronary angiography data.**Patients with different extent of coronary artery disease (CAD) presented by HF phenotype. Number of patients are shown within the circles, and total numbers with respect to phenotype above the circles, respectively.
CAD angiography data was missing in one patient (n=80).
1- or 2-VD: one or two-vessel disease; 3-VD: three-vessel disease; LMS: stenosis of the left main coronary artery

**
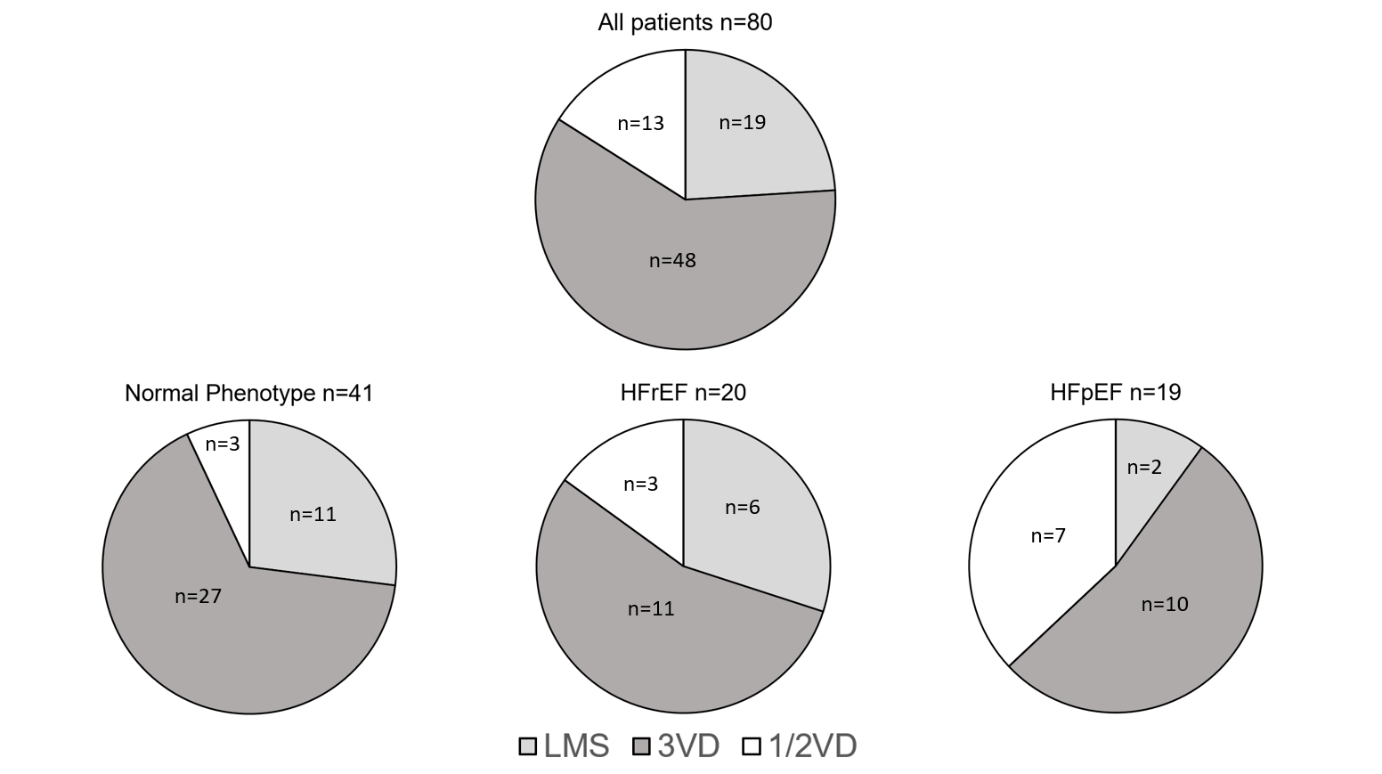
**

**Supplementary Figure 2.**Flow cytometry plots. All samples obtained in blood from coronary sinus.
**A**. Representative dot plot of extracellular vesicle gating (based on size and complexity) together with gates demonstrating beads with diameter 0.3, 0.5 and 0.9 μm, respectively.
**B** and **C**. Representative dot plot (combined 10 samples) of extracellular vesicles labeled with negative isotype controls (B; left) or anti-Troponin-T (C; right).
**D** and **E**. Representative dot plot (combined 10 samples) of extracellular vesicles labeled with negative isotype controls (D; left) or anti-Connexin-43 and anti-Troponin-T (E; right).


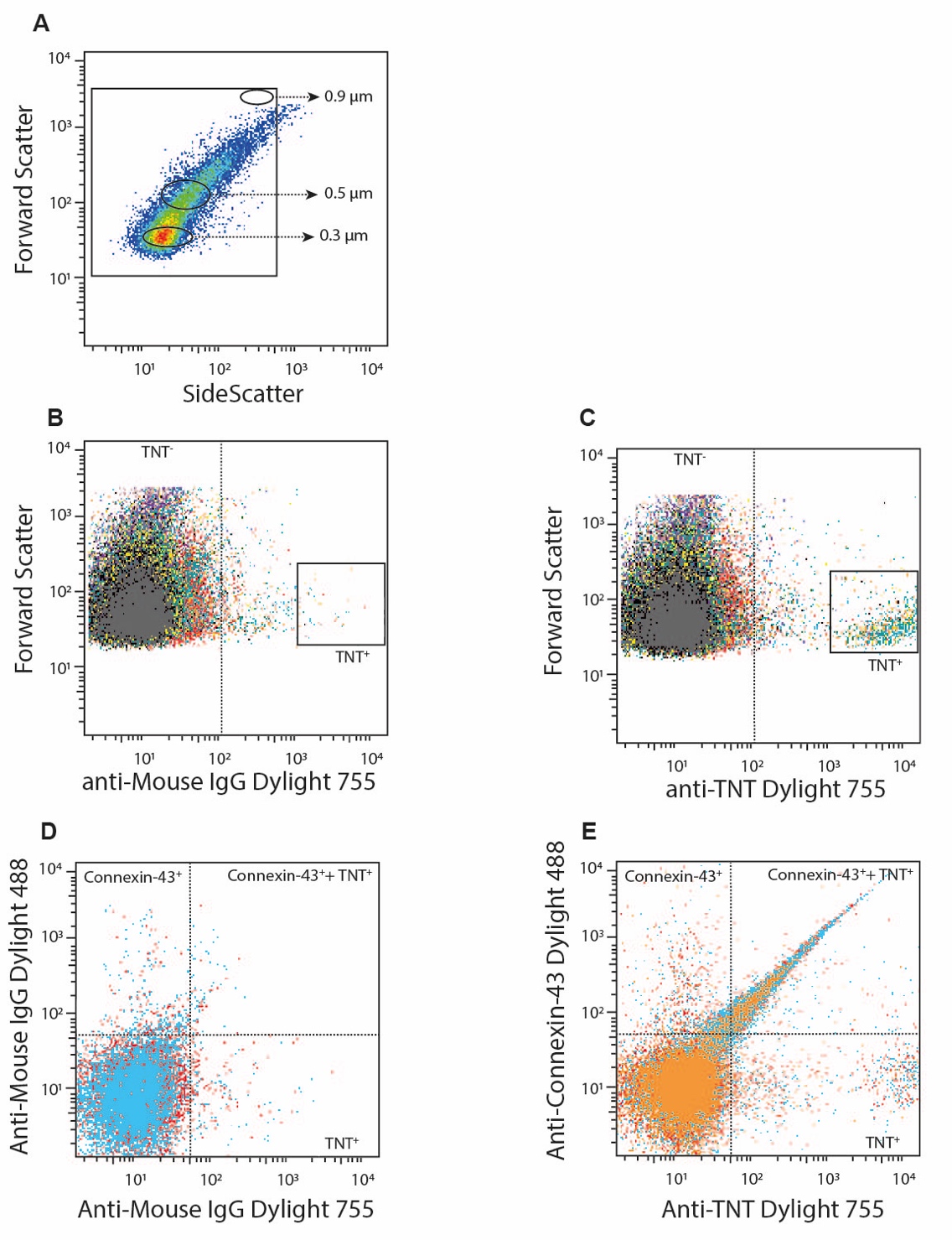


**Supplementary Figure 3.**Schematic overview of the different types of EVs investigated. The EVs were first identifed by size and complexity (supplementary Figure 2) and then different antigens carried by the EVs were detected using fluorescent labelled antibodes as described in the supplementary methods section and supplementary Figure 2. The antigens studied were: Connexin-43 - the most abundant gap junction molecule in cardiomyocytes but also present in various other cell types, Caveolin3 – a molecule mainly expressed in cardiomyocytes and skeletal muscle cells, Troponin T present in cardiomyocytes but also to some extent in skeletal muscle cells, N-cadherin present in cardiomyocytes but also smooth muscle cells and endothelial cells, VE-cadherin (CD144) from endothelial cells, or antigens reflecting inflammation (Myeloperoxidase [MPO] and Pentraxin3; no cell specific antigen studied for these vesicles). References regarding antigens are numbered (Roman numerals) in the Figure and full references given below the Figure.

**
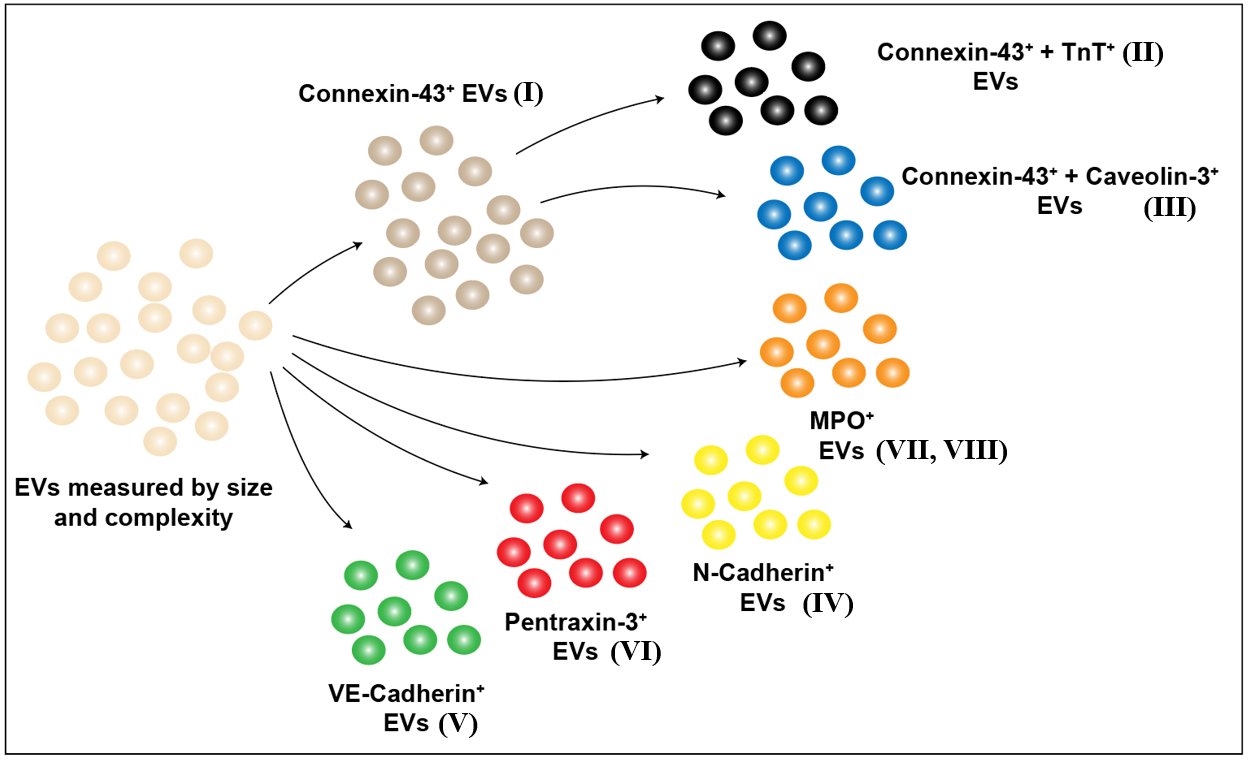
**

I. Peccoraro M et al. Role of connexin 43 in cardiovascular diseases. *Eur J Pharmacol* 2015; 768: 71-76
II. deFillipi CR, Damluji AA. At the crossroad between skeletal and cardiac muscle cells. Circulation 2022; 145: 1780-1783
III. Yin H et al. Caveolin proteins: a molecular insight into disease. *Front Med* 2016; 10: 397-404
IV. Resink TJ et al. Cadherins and cardiovascular disease. *Swiss Med Wkly* 2009; 139: 122-134
V. Vestweber D. VE-cadherin: the major endothelial adhesion molecule controlling cellular junctions and blood vessel formation. *Arterioscler Thromb Vasc Biol* 2008; 28: 223-232
VI. Falasca M et al. The Long Pentraxin PTX3: A Biomarker Spanning from Cardiovascular Disorders to Cancer. *J Mol Biomark Diagn* 2013; 4(2)
VII. Koeth RA et al. Myeloperoxidase in cardiovascular disease. *Adv Clin Chem* 2013; 62: 1-32
VII. Ndrepepa G. Myeloperoxidase – A bridge linking inflammation and oxidative stress with cardiovascular disease. *Clin Chim Acta* 2019; 493: 36-51

**Supplementary figure 4.**
EVs exposing Connexin-43+Caveolin-3 in samples from coronary sinus and radial artery.
Box plots indicate median, 25^th^ / 75^th^ percentile and 10^th^ / 90^th^ percentile. Wilcoxon signed rank test.


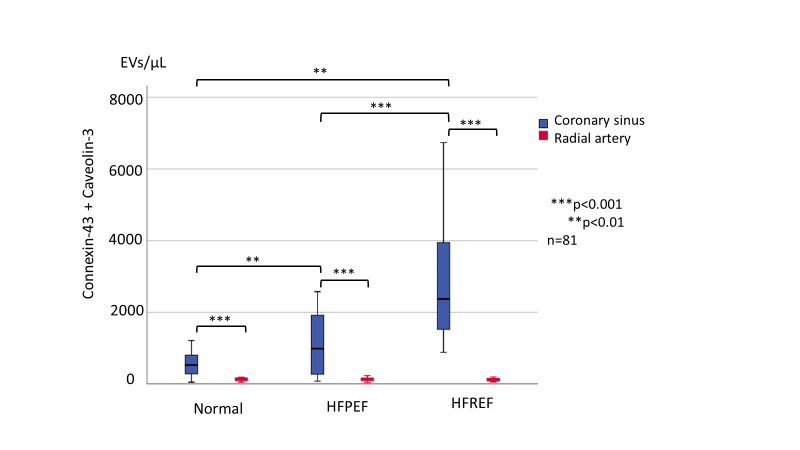


**Supplementary figure 5.**
EVs exposing Connexin-43+Troponin T (TnT) in samples from coronary sinus and radial artery. Box plots indicate median, 25^th^ / 75^th^ percentile and 10^th^ / 90^th^ percentile. Wilcoxon signed rank test.


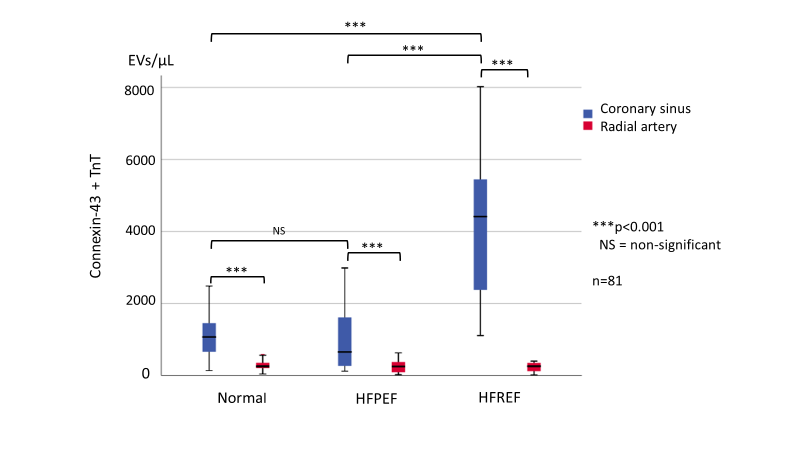


**Supplementary figure 6.**
EVs exposing VE-Cadherin in samples from coronary sinus and radial artery.
Box plots indicate median, 25^th^ / 75^th^ percentile and 10^th^ / 90^th^ percentile. Wilcoxon signed rank test.

**
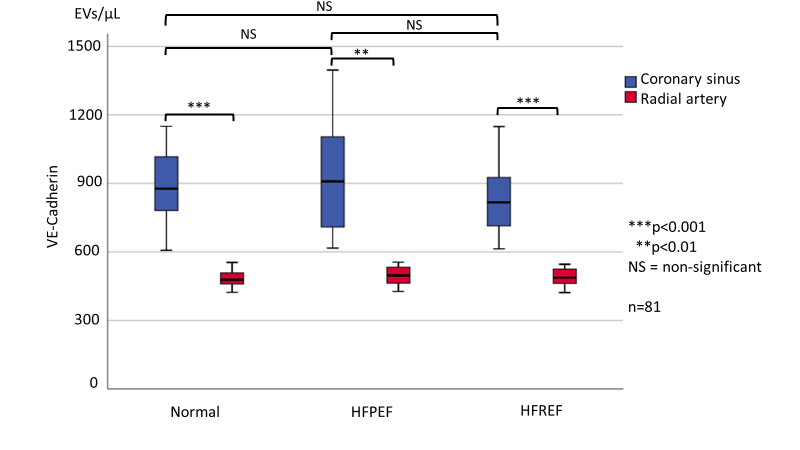
**

**Supplementary figure 7.**
EVs exposing Pentraxin-3 in samples from coronary sinus and radial artery.
Box plots indicate median, 25^th^ / 75^th^ percentile and10^th^ / 90^th^ percentile. Wilcoxon signed rank test.

**
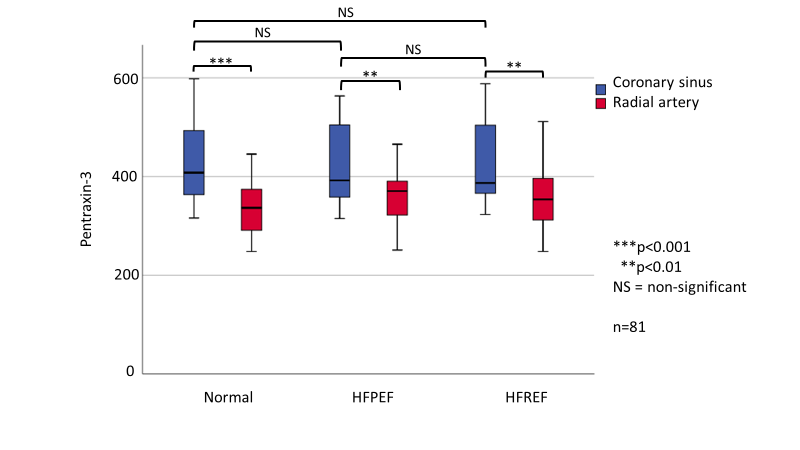
**

**Supplementary figure 8.**
EVs exposing Myeloperoxidase (MPO) in samples from coronary sinus and radial artery.
Box plots indicate median, 25^th^ / 75^th^ percentile and10^th^ / 90^th^ percentile. Wilcoxon signed rank test.

**
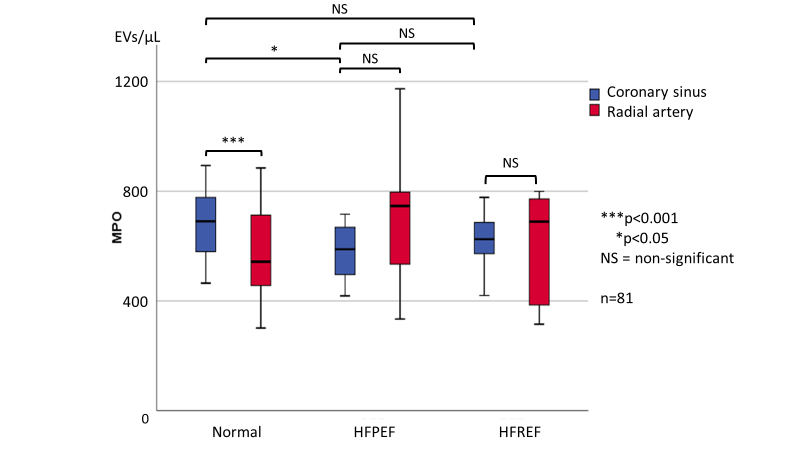
**

**Supplementary Table 1.**
Connexin-43/Caveolin-3 EV and Connexin-43/Troponin T EV concentrations (median and IQR; Vesicles/μL) in coronary sinus in relation to echocardiographic variables or NT-pro-BNP levels in plasma, divided in tertiles (median and IQR).
Kruskal – Wallis test was used to test statistical differences between tertiles.

|  | *Tertile 1* | *Tertile 2* | *Tertile 3* | *P-value* |
| --- | --- | --- | --- | --- |
| **LV GLS (%)** | -12.3 (3.0) n=27 | -16.9 (2.0)  n=28 | -20.0 (2.0)  n=26 | - |
| Con43/Cav3 | 1632 (1527) | 663  (603) | 434  (756) | P < 0.001 |
| Con43/TnT | 2258  (4160) | 1116  (1039) | 1039  (925) | P < 0.001 |
|  | | | | |
| **EF (%)** | 44 (8.0)  n=27 | 57 (2.0)  n=28 | 63 (4.0)  n=26 | - |
| Con43/Cav3 | 1632  (2074) | 519  (728) | 716  (1000) | P < 0.001 |
| Con43/TnT | 3193  (3777) | 952  (1204) | 858  (730) | P < 0.001 |
|  | | | | |
| **NT-pro-BNP (ng/L)** | 80 (75) n=26 | 222 (104)  n=28 | 1863 (2446)  n=26 | - |
| Con43/Cav3 | 456  (588) | 921  (959) | 1545  (1937) | P < 0.001 |
| Con43/TnT | 1088  (927) | 1220  (1122) | 2702  (4443) | P = 0.024 |
|  | | | | |
| **E/é** | 7.2 (1.4) n=29 | 8.9 (0.8)  n=25 | 11.8 (2.7)  n=27 | - |
| Con43/Cav3 | 571  (813) | 952  (1816) | 881  (1471) | P = 0.38 |
| Con43/TnT | 1108 (1196) | 1328  (1548) | 1439  (2120) | P = 0.25 |
|  | | | | |
| **LAVI  (mL/m^2^)** | 29.5 (5.0) n=27 | 35.1 (4.0)  n=27 | 49.0 (15)  n=27 | - |
| Con43/Cav3 | 546 (596) | 833  (1229) | 1856  (1490) | P < 0.001 |
| Con43/TnT | 1053  (848) | 1467  (1283) | 1501  (4735) | P = 0.16 |

**Supplementary Table 2**. Pharmacological treatment in patients with EVs measured.
Numbers and proportions (%) are shown.

|  | All patients | Patient category | | |
| --- | --- | --- | --- | --- |
|  |  | HFpEF | HFrEF | Normal |
| N | 81 (100%) | 19 (23%) | 20 (25%) | 42 (52%) |
| Antiplatelet agents | 59 (73%) | 14 (74%) | 13 (65%) | 32 (76%) |
| Oral Anticoagulants | 10 (12%) | 1 (5%) | 7 (35%) | 2 (5%) |
| Beta-blockers | 53 (65%) | 12 (63%) | 17 (85%) | 24 (57%) |
| ACE-inhibitors | 18 (22%) | 4 (21%) | 5 (25%) | 9 (21%) |
| Angiotensin receptor blockers | 22 (27%) | 4 (21%) | 10 (50%) | 8 (19%) |
| Furosemide | 6 (7%) | 3 (16%) | 3 (15%) | 0 |
| Mineralocorticoid antagonists | 3 (4%) | 1 (5%) | 2 (10%) | 0 |
| Statins | 66 (81%) | 13 (68%) | 19 (95%) | 34 (81%) |

**Supplementary table 3.**
Transcoronary concentrations gradients of extracellular vesicle (EV) types (EVs/μl) grouped by HF phenotype. Gradients (Δ) were calculated as concentration in coronary sinus *minus* concentration in radial artery.

|  | Δ Connexin43  + Caveolin | Δ Connexin43 + Troponin T | Δ N-Cadherin | Δ VE-  Cadherin | Δ MPO | Δ PTX3 |
| --- | --- | --- | --- | --- | --- | --- |
| All patients  n=81 | 702  (267 - 1486) | 1015  (438 - 1899) | -89  (-251-103) | 384  (266 - 520) | 99  (-92-245) | 69  (21 - 141) |
| HFpEF  n=19 | 930  (243 - 1807) | 305  (-50 - 1354) | -73  (-342 - 61) | 418  (212 - 618) | -56  (-241-186) | 33  (11 - 103) |
| HFrEF  n=20 | 2011  (1364 - 3886) | 4130  (1964 - 5339) | -199  (-291- -15) | 374  (203 - 473) | 15  (-126 - 193) | 69  (11 - 157) |
| Normal  n=42 | 391  (116 - 637) | 842  (438 - 1143) | -91  (-244 - 162) | 382  (290 - 506) | 164  (-3 - 331) | 81  (32 - 168) |
| p_1_ | 0.002 | <0.001 | 0.569 | 0.461 | 0.396 | 0.496 |
| p_2_ | 0.020 | 0.166 | 0.483 | 0.791 | 0.014 | 0.090 |
| p_3_ | <0.001 | <0.001 | 0.110 | 0.391 | 0.077 | 0.451 |

MPO = myeloperoxidase, PTX3 = Pentraxin-3,
Δ = differences in concentrations between coronary sinus and radial artery
Data are shown as medians and 25^th^ and 75^th^ percentiles.
P-values refer to comparison between groups, Mann-Whitney U-test.
p_1_ = HFpEF vs HFrEF, p_2_ = HFpEF vs Normal, p_3_ = HFrEF vs Normal.

**Supplementary table 4.**
Transcoronary concentrations gradients of extracellular vesicles (EV/μl) grouped according to coronary artery disease (CAD) severity based on preoperative coronary angiography. Gradients were calculated as concentration in coronary sinus (CS) *minus* concentration in radial artery (RA).

|  | Δ Connexin-43 + Caveolin-3 | Δ Connexin-43 + TnT | Δ N-Cadherin | Δ VE-Cadherin | Δ MPO | Δ PTX3 |
| --- | --- | --- | --- | --- | --- | --- |
| All  n=80 | 702  (267-1486) | 1015  (438-1899) | -89  (-251-103) | 384  (266-520) | 99  (-92-245) | 69  (21-141) |
| CAD stage 1  1-2 VD, no LM stenosis  n=13 | 72  (-41-1440) | 191  (-23-967) | -76  (-342-16) | 314  (123-487) | -241  (-350 - -63) | 17  (-19-108) |
| CAD stage 2  3 VD, no LM stenosis  n=48 | 749  (399-1789) | 1111  (614-1840) | -86  (-247-105) | 418  (312-592) | 118  (-78-253) | 71  (20-126) |
| CAD stage 3  LM stenosis  n=19 | 702  (238-1283) | 1141  (551-1964) | -156  (-293-24) | 356  (197-403) | 148  (9-219) | 87  (43-190) |
| P_1_* | 0.146 | 0.032 | 0.751 | 0.192 | 0.001 | 0.130 |
| P_2_* | 0.287 | 0.024 | 0.791 | 0.940 | 0.001 | 0.020 |
| P_3_* | 0.412 | 0.770 | 0.359 | 0.016 | 0.607 | 0.245 |

LM = left main artery disease; 1-2 VD = one or two-vessel (coronary artery) disease; 3 VD = three vessel (coronary artery) disease.
MPO = myeloperoxidase, PTX3 = Pentraxin-3,
Δ = differences in concentrations between coronary sinus and radial artery
Data are shown as medians and 25^th^ and 75^th^ percentiles.
P-values refer to comparison between groups, Mann-Whitney U-test.
*P_1_ = CAD stage 1 vs CAD stage 2; P_2_ = CAD stage 1 vs CAD stage 3; P_3_ = CAD stage 2 vs CAD stage 3.

**Supplementary Table 5**
Concentrations of extracellular vesicles (EVs) in plasma samples from coronary sinus in patients with and without atrial fibrillation, with and without diabetes, and in patients separated for sex.

|  | Atrial fibrillation | | p* | Sex | | p* | Diabetes | | p* |
| --- | --- | --- | --- | --- | --- | --- | --- | --- | --- |
|  | Yes | No |  | M | F |  | Yes | No |  |
|  | n=14 | n=67 |  | n=71 | n=10 |  | n=25 | n=56 |  |
| Connexin-43 +Caveolin-3, EV/μL | 1345 (1040-2711) | 792 (343-1366) | 0.012 | 908 (379-1737) | 687 (311-833) | 0.509 | 875 (355 - 2157) | 835 (368-1254) | 0.385 |
| Connexin-43 +TnT, EV/μL | 3878 (867-5653) | 1225 (620-1860) | 0.009 | 1225 (642-2194) | 1462 (1163-1687) | 0.381 | 1405 (760-2485) | 1233 (599-1861) | 0.360 |
| MPO, EV/μL | 652 (612-773) | 629 (570-736) | 0.450 | 651 (574-739) | 624 (579-725) | 0.886 | 664 (600-740) | 628 (570-731) | 0.440 |
| VE-Cadherin, EV/μL | 786 (664-917) | 898 (781-1036) | 0.030 | 879 (764-1022) | 923 (787-1043) | 0.625 | 903 (775-1043) | 878 (758-1008) | 0.563 |
| PTX3, EV/μL | 385(366-471) | 403 (364-498) | 0.067 | 396 (366-498) | 407 (357-476) | 0.590 | 406 (371-491) | 391 (362-499) | 0.347 |
| N-Cadherin, EV/μL | 476 (306-677) | 545 (389-724) | 0.072 | 536 (378-712) | 560 (355-738) | 0.242 | 480 (417-546) | 470 (388-533) | 0.519 |

TnT = Troponin-T, MPO = myeloperoxidase, PTX3 = pentraxin-3, M = male, F = female. Data shown as median and IQR. * P-values refer to comparison between groups, Mann-Whitney U-test.

**References**

1. Linde C, Eriksson MJ, Hage C, Wallen H, Persson B, Corbascio M, et al. Rationale and design of the PREFERS (Preserved and Reduced Ejection Fraction Epidemiological Regional Study) Stockholm heart failure study: an epidemiological regional study in Stockholm county of 2.1 million inhabitants. Eur J Heart Fail. 2016;18(10):1287-97.

2. Ponikowski P, Voors AA, Anker SD, Bueno H, Cleland JGF, Coats AJS, et al. 2016 ESC Guidelines for the diagnosis and treatment of acute and chronic heart failure – Web Addenda <https://www.escardio.org/static-file/Escardio/Guidelines/ehw128_Addenda.pdf2016>

3. Persson H, Donal E, Lund LH, Matan D, Oger E, Hage C, et al. Importance of structural heart disease and diastolic dysfunction in heart failure with preserved ejection fraction assessed according to the ESC guidelines - A substudy in the Ka (Karolinska) Ren (Rennes) study. Int J Cardiol. 2019;274:202-7.

4. Persson H, Lonn E, Edner M, Baruch L, Lang CC, Morton JJ, et al. Diastolic dysfunction in heart failure with preserved systolic function: need for objective evidence:results from the CHARM Echocardiographic Substudy-CHARMES. J Am Coll Cardiol. 2007;49(6):687-94.

5. Lang RM, Badano LP, Mor-Avi V, Afilalo J, Armstrong A, Ernande L, et al. Recommendations for cardiac chamber quantification by echocardiography in adults: an update from the American Society of Echocardiography and the European Association of Cardiovascular Imaging. J Am Soc Echocardiogr. 2015;28(1):1-39.e14.

6. Nagueh SF, Smiseth OA, Appleton CP, Byrd BF, 3rd, Dokainish H, Edvardsen T, et al. Recommendations for the Evaluation of Left Ventricular Diastolic Function by Echocardiography: An Update from the American Society of Echocardiography and the European Association of Cardiovascular Imaging. J Am Soc Echocardiogr. 2016;29(4):277-314.
